# Increase in body mass index during the COVID-19 pandemic among people who smoke: An analysis of multi-site electronic health records

**DOI:** 10.1371/journal.pgph.0001474

**Published:** 2023-04-11

**Authors:** R. Constance Wiener, Christopher Waters, Emily Morgan, Patricia A. Findley, Chan Shen, Hao Wang, Usha Sambamoorthi

**Affiliations:** 1 Department of Dental Public Health and Professional Practice, West Virginia University, Morgantown, West Virginia, United States of America; 2 Department of Dental Research, West Virginia University, Morgantown, West Virginia, United States of America; 3 Research Data Services, West Virginia University, Morgantown, West Virginia, United States of America; 4 Rutgers School of Social Work, New Brunswick, New Jersey, United States of America; 5 Department of Health Services Research, Penn State College of Medicine, Hershey, Pennsylvania, United States of America; 6 Department of Emergency Medicine, JPS Health Network, Fort Worth, Texas, United States of America; 7 Department of Pharmacotherapy, University of North Texas Health Science Center, Fort Worth, Texas, United States of America; University of Southampton, UNITED KINGDOM

## Abstract

The effects of the COVID-19 period among people who smoke (compared by sex) are largely unknown. The purpose of this study was to compare body mass index (BMI) increase among men and women who smoked during the pandemic. We used a retrospective longitudinal, observational study design of secondary data. We used electronic health records from TriNetX network (n = 486,072) from April 13, 2020-May 5, 2022 among adults aged 18–64 who smoked and had a normal BMI prior to the pandemic. The main measure was a change of BMI from < 25 to ≥25. Risk ratio was determined between men and women with propensity score matching. Overall, 15.8% increased BMI to ≥25; 44,540 (18.3%) were women and 32,341 (13.3%) were men (Risk Ratio = 1.38, 95% CI: 1.36, 1.40; *p* < .0001). Adults with diabetes, hypertension, asthma, COPD or emphysema or who were women, were more likely to develop BMI≥25 during the pandemic. Women who smoked were more likely to have an increase in BMI than men who smoked during the COVID-19 period.

## Introduction

During the early phase of the coronavirus disease 2019 (COVID-19) pandemic, emergencies were declared which included self-quarantines, travel restrictions, and lock downs. In the U.S., residents were encouraged to stay indoors, wear masks, wash hands frequently, and keep a social distance of six feet between people. Multiple waves of COVID-19 resulted in prolonged high levels of stress associated with the seriousness of the disease. Anecdotal reports indicated that stress, anxiety, depression, less physical activity, and more sedentary activities resulted in weight gain, described as “quarantine 15” [1].

For example, in a survey of people with a mean body mass index (BMI) of 27 (n = 111), 22% self-reported gaining 5–10 pounds during the pandemic [1]. Respondents in another survey (n = 764) reported mean weight gain of 0.62 kg [2]. In an anthropometric study of weight and COVID-19 (n = 11,534), the mean weight increase for men was 3.41 kg; while the mean weight increase for women was lower, at 3.09 kg; however, the weight gain did not correspond to a significant difference in BMI increase between men and women [3]. The number of people developing BMI≥25 during the COVID-19 pandemic was particularly concerning due to the link between obesity and increased COVID-19 morbidity/mortality [3–6] through the effects of adipose tissue on the immune system such as reducing the response to antiviral agents through poor T-cell and macrophage actions; chronic activation of the immune system; and altered secretion of inflammatory mediators [4]. Additionally, during the 2009 H1N1 pandemic, obesity was a risk factor for severity, hospitalization, increased risk of transmission and mortality, therefore, extrapolating to COVID-19, obesity is a concern with similar poor health outcomes [4]. Examining overweight/obesity *and smoking* during the COVID-19 pandemic are important as:

1. smoking is associated with risk of central obesity [7];
2. smoking cessation is associated with weight gain [8]; and
3. smoking increases morbidity/mortality with COVID-19 [9,10].

Mechanistically, smoking increases COVID-19 morbidity/mortality by the direct toxic effects of smoking injury to the nose and lower airway tissue enhancing infectivity; and, an antiviral inflammatory cascade that increases the virus’s pathogenesis [11]. In addition, disturbances in the angiotensin-converting enzyme 2, or in the renin-angiotensin system may be altered with smoking [11], resulting in inadequate lung protection from infections [11].

The purpose of the present research was to examine increases in BMI during the pandemic (April 13, 2020 to May 5, 2022) among people who smoked and had a normal BMI prior to the pandemic. This observational study assesses the change in BMI with repeated observations over two time periods using harmonized electronic health records (EHR).

## Methods

### Ethical statement

The research received acknowledgment from the West Virginia University Institutional Review Board as non-human subject research.

### Study design

The research had a longitudinal, observational study design of secondary data.

### Data source

The data were extracted from TriNetX (Cambridge, MA), a system in which real-time, de-identified healthcare organization (HCO) data are available to researchers. Before distribution, the data are coded for anonymity and are extracted from structured EHR data and narrative texts [12]. Data analysis is available through TriNetX cloud-based internal custom platform from Java 1.9.0_171, R 3.44 (R core Team, Vienna, Austria) and Python 3.6.5 [13]. This population-based study included data of EHR of all who visited one of the 58 reporting HCO’s.

### Inclusion/Exclusion criteria

The inclusion criteria at baseline were records with complete data on biological sex (male/female), age (with restrictions to ≥18 years and <65 years), that the EHR indicated current smoking (ICD-10-CM codes F17 or Z72.0) and had a pre-existing BMI ≥18 to <25 on March 13, 2020. Records with a BMI≥25 before March 13, 2020 were excluded. Additional inclusion criteria were that the participant had a follow-up appointment in which BMI was determined by May 5, 2022, and that the participant reported that they had continued to smoke.

### Measures

The outcome variable was moving to the category BMI≥25 from lower BMI between April 13, 2020 to May 5, 2022 (yes, no). Biological sex was the main independent variable (male, female) of interest for the comparison.

### Propensity score matching variables

The TriNetX system provides characteristics and signal strengths to determine if matching is statistically warranted. Standard differences are provided to determine matching success with <25% standard difference recommended on matched variables. For this study, several epidemiological factors were matched: hypertensive disease, diabetes mellitus, asthma, emphysema, and other chronic obstructive pulmonary diseases (COPD), race and age.

### Time frame

The study included all available EHR that excluded BMI≥25 before the March 13, 2020 baseline. The index date for the onset of BMI≥25 was April 13, 2020. The development of BMI≥25 was analyzed with a short-term horizon, the pandemic period from April 13, 2020 to May 5, 2022 [14].

### Statistical analysis

The internal TriNetX software was used for change in BMI category and risk of BMI≥25 during the pandemic using logistic regression analyses.

## Results

Before matching, the study sample included 531,416 records of people who smoked in April 2020. There were 248,604 (46.8%) females and 282,812 (53.2%) males. The mean age was 36.75 years (standard deviation, 13 years). After matching, all variables had standardized differences <0.06 (Table 1).

**Table 1:** Creating the matched cohorts.

	Total before Pandemic (no matching)	Percent of cohort	Total before pandemic (matching)	Percent of cohort
**Hypertension**				
** Female, yes** ^**a**^	7,753	3.12%	7,659	3.15%
** Female, no**	240,851	96.88%	235,377	96.85%
** Male, yes** ^**a**^	11,605	4.10%	7,573	3.12%
** Male, no**	271,207	95.90%	235,463	96.88%
**Yes, Diabetes**				
** Female, yes** ^**a**^	3,903	1.57%	3,802	1.56%
** Female. no**	244,701	98.43%	239,234	98.44%
** Male, yes** ^**a**^	5,381	1.90%	3,629	1.49%
** Male, no**	277,431	98.10%	239,407	98.51%
**Asthma**				
** Female, yes** ^**a**^	4,247	1.71%	3,327	1.37%
** Female, no**	244,357	98.29%	239,709	98.63%
** Male, yes** ^**a**^	3,467	1.23%	3,193	1.31%
** Male, no**	279,345	98.77%	239,843	98.69%
**Emphysema**				
** Female, yes** ^**a**^	512	0.21%	509	0.21%
** Female, no**	248,092	99.79%	242,527	99.79%
** Male, yes** ^**a**^	908	0.32%	519	0.21%
** Male, no**	281,904	99.68%	242,517	99.79%
**COPD**				
** Female, yes** ^**a**^	2,310	0.91%	2,140	0.88%
** Female, no**	246,294	99.09%	240,896	99.12%
** Male, yes** ^**a**^	2,521	0.89%	2,115	0.87%
** Male, no**	280,291	99.11%	240,921	99,13%

^a^Items matched were **positive** responses to: Hypertension, diabetes, asthma, emphysema, and COPD. Age and sex were also matched.

Overall, 15.8% moved from the normal BMI category to BMI≥25; 44,540 (18.3%) women and 32,341 (13.3%) men (p < .0001) (Table 2).

**Table 2:** Change from normal BMI category to overweight/obese category, pre-pandemic to May, 2022.

	Total before pandemic	BMI changed to ≥25	Percent changed	p-value	Total before pandemic (matching)	BMI changed to ≥25	Percent changed	p-value
**Sex**				< .0001				< .0001
**Female**	248,604	45,536	18.3		243,036	44,540	18.3	
**Male**	282,812	38,378	13.6		243,036	32,341	13.3	

Two logistic regression analyses were conducted on moving to BMI≥25 from normal BMI. In unmatched logistic regression, the risk ratio (RR) of BMI≥25 for females was 1.35 (95% Confidence interval [CI]: 1.33, 1.37; *p* < .0001). The matched RR of BMI≥25 for females was 1.38 (95% CI: 1.36, 1.40; *p* < .0001) (Table 3).

**Table 3:** Risk ratios for biological sex on BMI ≥ 25 during the COVID-19 pandemic risk ratio before propensity score matching risk ratio after propensity score matching.

	RR and 95%CI	p-value	RR and 95% CI	p-value
**Men**	**Reference group**		**Reference group**	
**Women**	**1.35 (1.33, 1.37)**	**< .0001**	**1.38 (1.36, 1.40)**	**< .0001**

Abbreviations: RR, risk ratio; CI, confidence interval Analysis conducted on May 4, 2022.

## Discussion

We compared the increase in BMI from normal BMI to BMI≥25 during the pandemic among people who smoked. Overall, 15.8% developed BMI≥25. Women were 38% more likely than men to move from normal BMI to BMI≥25. Two substantial percent changes to BMI≥25 were among people who had diabetes (39.2% developed BMI≥25) and people who had asthma (37.3% developed BMI≥25). These results are concerning as, in addition to COVID-19, there are long-term consequences of BMI≥25 and diabetes, as well as BMI≥25 and asthma exacerbations.

Several factors are potentially associated with the findings. The COVID-19 quarantine, fear, economic downturn, and closures likely increased stress and that psychopathology could have influenced eating behavior [15]. Researchers indicated many symptoms of psychopathology did not vary by gender during that time, but eating pathology was increased in women (Beta = -0.013 [95% CI: -0.042, -0.004; *p* = 0.049) [15].

Previous researchers indicated that adults who smoked were more likely to weigh less than adults who did not smoke [7]. Nicotine is considered to increase metabolic rate, decrease metabolic efficiency, decrease caloric absorption and increase the acute anorexic effect [7]. Observing a change in BMI from normal BMI to BMI≥25 in people who smoke carries additional concerns for potential COVID-19 outcomes and other health outcomes.

### Strengths and limitations

This study’s strength is the use of national, real-time data from 58 HCO’s with the ability to match and determine BMI outcomes. The study included diverse HCOs across the U.S. The study findings have implications for COVID-19 prognosis as other researchers have indicated that BMI≥25 is a significant factor for severe COVID-19 [4,16].

A study weakness is the use of data that were designed for clinical purposes rather than research. For example, there is a lack of race/ethnicity data in some HCO EHR as race/ethnicity may not have been of clinical relevance to that HCO. The propensity score matching approach assumes “no unmeasured confounders.” However, the database did not have information on variables that are not routinely collected in clinical visits such as physical activity and calorie intake. The observed BMI change could have resulted from inactivity, increased calories, or a combination of both during the pandemic.

There is also the lack of information in the EHR’s concerning the number of cigarettes smoked on a daily basis and historical pack-years smoked. Other limitations are that we did not match/control for medications; or match for other chronic conditions that may affect BMI beyond hypertension, diabetes, asthma, emphysema, and chronic obstructive pulmonary disease. Using the TriNetX platform for analysis, we were limited by the query procedures available in the platform. Although it is important to examine biological sex-specific effects as conclusions from one sex cannot be assumed for the other [17], the emphasis of the study was change BMI≥25 through the factors of smoking and sex. We recognize that the development of overweight/obesity requires time and there are factors other than the pandemic that may have influenced weight gain in the study’s time window (0.08 year to 2.16 years from declaration of COVID restrictions in the U.S.). Furthermore, the baseline measurement time window can vary from a minimum of xx to a maximum of xx.

### Public health considerations

Smoking and BMI≥25 increase COVID-19 morbidity/mortality, therefore it is important to have the epidemiological information for efforts in reducing COVID-19 harm. It is important to address the risks associated with BMI≥25 and COVID-19 as well as guiding the public toward a normal BMI and tobacco cessation. The messaging needs to be person-centered and appropriate to improve overall whole health.

### Conclusion

Among people who smoked and were normal BMI before the pandemic, people with diabetes, hypertension, asthma, COPD or emphysema or women were more likely to develop BMI≥25 during the pandemic.
